# Supplementary material for: Hybrid machine learning for real-time prediction of edema trajectory in large middle cerebral artery stroke
Source: NPJ Digit Med. 2025 May 17;8:288. doi: 10.1038/s41746-025-01687-y (PMC12084630; doi:10.1038/s41746-025-01687-y)
Supplement: Supplementary file 1 — Supplementary Information [file 41746_2025_1687_MOESM1_ESM.pdf]

SUPPLEMENTARY INFORMATION

1 Review of Existing Prediction Models

We present five existing models for predicting malignant MCA infarction or malignant brain edema. All of the reviewed models use logistic regression to make static predictions of late-stage patient outcomes, and are therefore limited in their applicability to real-time medical decision-making. The five existing models are summarized in Supplementary Table 1.

2 Definitions of Key Extracted Variables

2.1 Stroke Characteristics

Last seen well date and times were identified via clinical notes. Last seen well was defined as the last time before a patient experienced a substantial deficit as indicated by the NIHSS (symptoms of numbness, tingling, or other symptoms that would result in NIHSS < 3 were not taken as the time of last seen well). Instances where last seen well lacked exact times or only referenced time were labeled as "inferred." If last-seen well occurred prior to an in-hospital surgery, the surgery time was identified. If no surgery start time was identified, surgery was assumed to be at 7:00AM. If last seen well referred to "last night before bed," with no exact time, then last seen well was assumed to be 10:00 PM. All last-seen well data underwent supervisor review (CJO).

NIHSS was extracted from history and/or physical exam. In cases where multiple NIHSS were reported, the last one (after any procedures took place) was used. NIHSS scores were documented via clinical notes. For patients who underwent medical or mechanical thrombolytic procedures, we prioritized the NIHSS recorded after intervention.

Vessel Occlusion location: All CT Angiograms were reviewed and classified as a vessel occlusion based on radiology reports. The most proximal vessel involved (Internal Carotid Artery, M1, M2, M3/M4) was used for the vessel occlusion location. The anterior cerebral artery was also identified as occluded or not. In instances in which reports described vessel occlusion but did not explicitly name vessel occluded, M1 was assigned if occlusion was described to begin prior to bifurcation/trifurcation.

2.2 Laboratory and Vital Sign Measurements

Laboratory and vital sign values were obtained through the structured Research Patient Data Registry/Clinical Data Warehouse database. The first value present within eight hours before and up to thirty-six hours after presentation date time was used, accounting for labs that occurred prior to the first radiographic scan and/or clinically determined presentation date time. The mean time from presentation to first laboratory value in the derivation cohort was 0.31 hours (approximately 19 minutes), with a standard deviation of 17.9 hours. Laboratory values taken prior to presentation time are due to presentation time definition—labs in the emergency department occasionally predated official "presentation time." Vital signs at presentation were derived from a chart review of the History and Physical. On average, vital signs data were updated every 51.0 hours (standard deviation 38.9 hours) and laboratory data were updated every 64.1 hours (standard deviation 40.3 hours) within the first week after presentation.

To address clearly erroneous lab or vital sign errors, each longitudinal measurement was assigned a Z-score based on the data distribution across the entire cohort. Measurements with an absolute Z-score of three or greater, or those showing a 25% change from a preceding value, were flagged as a potentially erroneous measurement. The principal investigator reviewed each patient's flagged laboratory/vital sign trajectories within the context of their hospital course to determine which outliers were valid and which needed to be removed. Temperature values recorded in Celsius (30°s-low 40°s) were converted to Fahrenheit. Other abnormally low-temperature values, such as 78°F, were removed. One sodium value of 102 mEq/L was removed after chart verification. All glucose values $\geq$1000 mg/dL and one glucose measurement of 866 mg/dL were removed after chart verification.

2.3 Radiographic Features

The population's radiographic features were derived using a Natural Language Processing algorithm that screens for patients with acute MCA stroke identified via ICD-9, ICD-10 codes [1]. Patients with large acute MCA stroke were confirmed to be $\geq$ 1/2 of the MCA territory by designated, trained M.D members of the research team (SC, CJO). Images included in $\geq$1/2 MCA territory had to have either full superior or inferior MCA division involvement or include sufficient volume (estimate of >70ccs) of both superior/inferior or deep structures. All indeterminate images underwent secondary review (CJO). A random sampling of our 30 patients using an ABC/2 method [2] demonstrated that median

volume was 122 cc. Below we outline the detailed process we applied to characterize each of the included radiographic features:

- *Stroke size*: $\geq$1/2 MCA territory was determined by visual estimate of a trained M.D. and underwent second review. Indeterminate images were reviewed for consensus by three authors.
- *Midline shift* was measured by a trained member of the team using imaging viewer software [Client Outlook, eUnity Diagnostic Viewer, version 6.10.2-489, for MacOs and PACS web viewer] by navigating to the level of the septum pellucidum at the slice of maximum MLS. The reviewer created a line connecting the attachment of falx cerebri anteriorly and the occipital protuberance. Windows were set at W:30 L:30. Distance between the midline and the septum pellucidum both the lateral and medial boundaries was measured by adding a line perpendicular to the midline. MLS used in the analysis was the average distance (mm) from the measurements of the lateral and medial boundaries. A blinded assessment of >10% of the data found the mean error was 0.19mm between the MLS reported in the radiographic reports and manual measurements.
- *Pineal gland shift* was measured at the level of the pineal gland, at the slice where the pineal gland shift was at its maximum. A line from the midline to the point of maximum and minimum deviation was taken, and averaged, to best obtain the deviation from the center of the pineal gland. Mean error between MD reviewers was 0.36mm.
- *ASPECTS*, or the Alberta Stroke Programme Early CT Score, divides the brain parenchyma into 10 separate non-overlapping regions, three regions at the level just rostral to the ganglionic structures and four at the level of the thalamus [3]. ASPECTS of 10 implies the brain parenchyma shows no evidence of ischemia, only at the two levels that the predetermined regions are examined. ASPECTS of 0 implies that all the predetermined areas have been affected by ischemia of the brain parenchyma, hypodensity, loss of cortical ribbon, and/or sulcal effacement. ASPECTS were generated using 5mm axial slices and set to variable window widths. The window, as described by Lev et al. [4], for optimal ischemic lesion identification is preferably set to W:30 L:30, or alternatively 40/40. As in Pexman et al. [5], patient positioning was reviewed to determine if the eyes were at the same level in the axial view. If >10% of each area had evidence of hypodensity or sulcal effacement, that area was marked as affected by ischemia and one point was deducted from the total score [3]. A review of 10% of scans showed a percent agreement between the two reviewers of 96.4% for dichotomous ASPECTS categories (10-8, 7-0), and 83.9% for high, medium, low ASPECTS (10-8, 7-4, 3-0). Cohen's Kappa was 0.647 for ASPECTS continuously.
- *Hemorrhage* was manually inspected according to ECASS II criteria. Petechial Hemorrhage was determined to be HI1 or HI2, and Parenchymal Hemorrhage PI1 or PI2 [6]. Percent agreement between trained team members (SC, CJO) on a 10% sample was 92.5%.
- *Cerebral atrophy* was recorded based on visual estimation of the ratio of sulcus to gyrus depth on the non-infarcted side and the ratio of the width of the caudate to the brain. A trained researcher evaluated admission non-contrast CT scans to determine the overall level of atrophy in the brain. Atrophy characterizations were categorized into no/mild atrophy (0) and moderate to severe atrophy (1). The percent agreement was 88%.
- *Basal Cistern Effacement* was determined (present/absent) in axial slices at the level of the orbitomeatal line after reviewing consecutive slices for evidence of partial or complete reduction of the basal cisterns space. Images were reviewed for consensus by three authors.

3 Missing Data & Frequency of Measurement

The derivation cohort was associated with a higher degree of missing static data compared to the external validation cohort, especially in vital signs at admission and in collateral scores, first MLS value, HbA1c, and osmolality. The number and percentage of patients in both cohorts with missing information regarding the static variables are presented in Supplementary Table 2. Our analysis also showed that vital signs were recorded more frequently at the Boston Medical Center, while laboratory data and radiographic variables were updated more frequently in the Massachusetts General Brigham cohort. These patterns may reflect differences in laboratory and scanning capacity across the two sites. However, we also note that the Massachusetts General Brigham cohort has a high degree of missingness in vital signs data, with fewer than 30% of all hourly observations having an updated vital signs measurement in the preceding 8 hours. This is because longitudinal vital sign data was not as commonly available prior to 2015. Supplementary Table 3 reports the proportion of patient-hour observations in the first week of hospitalization with an updated dynamic variable measurement in the past 8 hours, and Supplementary Table 4 presents the average time between updated measurements of dynamic variables within the first week of hospitalization across cohorts.

4 Hospitalization Characteristics Across Cohorts

To further compare the relative severity of mass effect after infarct across sites, we present the breakdown of patients in each cohort by maximum MLS class reached over the course of their hospitalization in Supplementary Table 5. No statistical significance was found between the proportion of patients in each maximum MLS class across the cohorts. We also describe key characteristics of hospitalization (e.g. length of stay, scanning frequency, and maximum MLS measured) for both cohorts with patients separated by MLS class at initial scan in Supplementary Table 6. Notably, the length of time between successive CT scans over the first week of hospitalization is higher (lower scanning frequency) for patients with higher MLS on admission. We also find that patients with higher initial MLS have a higher maximum MLS measurement, as would be expected.

5 Machine Learning Model Specification

5.1 HELMET Model Features

We report a breakdown of each of the features used in our HELMET models. Supplementary Table 7 describes the features used in the 24-hour prediction model while Supplementary Table 8 describes the features used in the 8-hour prediction model. All date-time features are represented as number of hours after last seen well (e.g. if the patient was last seen well at 12:00 and the first MLS occurred at 18:00, the value of *firstmlsdt_time_censored* would be 6). Time censored variables are assigned a place holder value until the time of occurrence to prevent future information from being leaked into the present (e.g. if firstmlsdt_time_censored was 6, then the first five patient-hour observations would be set to -10).

5.2 Comparison of XGBoost to Random Forest Models

We also tested Random Forest (RF) models [7] as part of our initial exploration (prior to adding the large language model-derived features). The Random Forest algorithm is an ensemble algorithm that creates multiple weak learner decision trees [7]. We once again assessed performance on the derivation dataset with five-fold cross-validation. XGBoost and RF achieved roughly equivalent performance, with a mean area under the receiver operating characteristic curve (AUROC) across the four classes of 0.86 (±0.017 for RF, ±0.0083 for XGBoost) for predicting 24-hour window maximum MLS, and 0.86 (±0.0066 for RF, ±0.0059 for XGBoost) for predicting 8-hour window maximum MLS. The relative similarity in the performance of XGBoost and RF models is consistent with their relatively similar ensemble tree architectures. In both settings, both models significantly outperformed the logistic regression baseline ($p<0.001$ on all tests for AUROC and AUPRC).

5.3 Final Tuned Hyperparameters

Using a combination of cross-validation within runs and Bayesian updating across successive model training runs, we tuned the model hyperparameters and non-transition observation weights for both the HELMET-8 and HELMET-24 models. The final values for both models are presented in Supplementary Table 10.

5.4 Parameter & Structure Sensitivity Analyses

We conducted a number of sensitivity analyses to improve the performance of the model. First, we tested the inclusion of a sample weighting parameter which reduced the relative importance of samples where the target MLS class was the same as the patient's current MLS class (in order to further prioritize training on the more ambiguous cases of patient class transition). Results showed that a relative importance reduction of 0.4-0.7 yielded increased filtered performance at the expense of some overall, unfiltered performance. Next, we tested adding the class probabilities from the average transition kernel as features and adding the class probabilities as predicted by the linear regression model (discussed below). Neither of these feature additions led to any significant performance improvements. Finally, we tested reducing the feature space by removing the lowest-ranked features from the feature importance values of the initial model. Reducing the feature space to approximately 75 total features yielded increased performance, with further feature reductions being detrimental. However, any attempts at feature reduction proved detrimental after the addition of the large language model class predictions to the feature space.

6 Model Performance

6.1 Large Language Model Performance

We report the modified AUROC metric for the multi-class classification task as defined in Section 4.7. Using derivation patient radiology reports, the large language model classifiers achieved AUROC scores of 72.23% for the 8-hour task, 67.25% for the 24-hour task, and 91.10% for the 36-hour task when tested on the derivation cohort. Applying the fine-tuned models to the external validation cohort, the large language model performance dropped to 48.04%, 50.13%, and 50.24% on the 8-hour, 24-hour, and 36-hour tasks respectively.

6.2 Complete Performance Metrics

In a filtered dataset using only prediction windows in which the patient's MLS class changed (the "filtered" task), the models result in a mean filtered AUROC of 94.1% (95% CI [92.3%, 96.0%]) for the 24-hour window and 76.2% (95% CI [66.6%, 85.8%]) for the 8-hour window on the derivation dataset. On the external validation dataset, the models result in a mean filtered AUROC of 70.7% (95% CI [61.1%, 80.3%]) for the 24-hour window and 92.1% (95% CI [88.5%, 95.7%]) for the 8-hour window. The filtered performance metrics show that the model performs well both when the patient is expected to remain in the same state and when the edema severity is changing. The complete unfiltered and filtered performance metrics, along with 95% confidence intervals are reported in Supplementary Table 11.

Due to the highly time-dependent nature of edema trajectory, we also calculated average AUROC across various periods of hospitalisation for each patient. These periods were defined based on hours since last seen well as <24 hours, 24-48 hours, 48-96 hours, and ≥ 96 hours. AUROC scores for each model across the two tasks and disaggregated by hospitalization period are presented in Supplementary Table 12. Finally, we also present mean AUROC scores disaggregated by patient insurance status in Supplementary Table 13.

6.3 Receiver Operating Characteristic Curves and Precision-Recall Curves

The receiver operating characteristic (ROC) curves for both models and both patient cohorts are presented in Supplementary Figure 1. Similarly, the precision-recall curves (PRC) for both models and both cohorts are presented in Supplementary Figure 2.

6.4 Shapley Additive Explanation Plots

SHapley Additive exPlanation (SHAP) values are derived from cooperative game theory, originally developed to fairly distribute payouts among players based on their contributions to an overall outcome. In the context of machine learning, SHAP values quantify the contribution of each feature to the prediction by simulating the model's behavior when each feature is included or excluded from the input data. Specifically, SHAP values represent the average marginal contribution of a feature across all possible combinations of feature subsets, ensuring that the contributions are fairly attributed in a manner consistent with the axioms of efficiency, symmetry, and additivity. SHAP plots indicate the aggregate influence of individual features on assigned classification categories. Each sub-bar's length indicates the mean SHAP value per class, highlighting the relative strength of a feature's influence on the prediction class outcome. The features are organized by significance, with the most crucial ones positioned at the top. Supplementary Figure 3 shows SHAP bar plots for the ensemble learning models.

7 Model Performance on a Binary Classification Task

An MLS of $\geq$5mm is commonly used in clinical practice as a signal of severe edema [8]. To evaluate the robustness and generalizability of our methodology, we evaluated the HELMET framework on a simplified binary classification task. This experiment benchmarks the HELMET models against the baseline EDEMA regression models in a more conventional target. This set of models focus on estimating whether the maximum MLS within the prediction window would exceed the 5mm threshold.

The resulting binary classification models were constructed following the HELMET framework, using the same multimodal input features and model architecture described in the Section 4 of the main manuscript. The only change was to the outcome definition: instead of predicting four discrete MLS classes, the models were trained to classify whether future MLS would be below or above the 5mm threshold. Corresponding EDEMA baseline models were also retrained for the binary task using the same respective inputs. Performance metrics for both model families—across derivation and external validation cohorts, 8-hour and 24-hour horizons, and both overall and filtered datasets—are reported in Supplementary Table 14.

Across both prediction horizons and cohorts, the HELMET models consistently achieved higher or comparable AUROC, AUPRC, and accuracy values compared to the EDEMA baselines. The overall AUROC scores for the binary task ranged from 88.4–91.0% for HELMET models, compared to 63.5–87.0% for the EDEMA baseline models. Improvements were especially notable in the external validation cohort, where the HELMET models demonstrated high predictive performance with lower variance, indicating improved generalizability. Additionally, performance on the filtered subsets—observations where MLS class transitioned across the threshold—was markedly better for HELMET models, suggesting superior sensitivity to clinically relevant changes.

The primary HELMET-8 and HELMET-24 models were based on more granular, four-class MLS targets (0mm, 0–3mm, 3–8mm, >8mm) to reflect a more nuanced progression of edema severity over time. The success of the HELMET framework on the simpler binary task provides additional evidence regarding the robustness and adaptability across a spectrum of prediction problems. The wider performance improvement over baseline observed in the main analysis of the multiclass prediction problem as compared with the smaller difference in overall performance observed for this binary prediction problem highlights the power of existing regression models for simpler problems and underscores the usefulness of our hybrid ensemble learning approach for more granular prediction.

8 Reporting Checklist

We followed the updated Transparent Reporting of Multivariable Prediction Models for Individual Prognosis or Diagnosis (TRIPOD+AI) guidelines in the reporting of our study [9]. The completed TRIPOD+AI checklist can be found in Figure 4.

References for Supplementary Information

1. Ong, C. J. *et al.* Machine learning and natural language processing methods to identify ischemic stroke, acuity and location from radiology reports. *PloS one* **15,** e0234908 (2020).
2. Sims, J. R. *et al.* ABC/2 for rapid clinical estimate of infarct, perfusion, and mismatch volumes. *Neurology* **72,** 2104–2110 (2009).
3. Barber, P. A., Demchuk, A. M., Zhang, J. & Buchan, A. M. Validity and reliability of a quantitative computed tomography score in predicting outcome of hyperacute stroke before thrombolytic therapy. *The Lancet* **355,** 1670–1674 (2000).
4. Lev, M. H. *et al.* Acute stroke: improved nonenhanced CT detection—benefits of soft-copy interpretation by using variable window width and center level settings. *Radiology* **213,** 150–155 (1999).
5. Pexman, J. W. *et al.* Use of the Alberta Stroke Program Early CT Score (ASPECTS) for assessing CT scans in patients with acute stroke. *American Journal of Neuroradiology* **22,** 1534–1542 (2001).
6. Hacke, W. *et al.* Randomised double-blind placebo-controlled trial of thrombolytic therapy with intravenous alteplase in acute ischaemic stroke (ECASS II). *The Lancet* **352,** 1245–1251 (1998).
7. Breiman, L. Random forests. *Machine Learning* **45,** 5–32 (2001).
8. Wijdicks, E. F. *et al.* Recommendations for the management of cerebral and cerebellar infarction with swelling: a statement for healthcare professionals from the American Heart Association/American Stroke Association. *Stroke* **45,** 1222–1238 (2014).
9. Collins, G. S. *et al.* TRIPOD+AI statement: updated guidance for reporting clinical prediction models that use regression or machine learning methods. *BMJ* **385.** eprint: `https://www.bmj.com/content/385/bmj-2023-078378.full.pdf`. `https://www.bmj.com/content/385/bmj-2023-078378` (2024).
10. Shimoyama, T. *et al.* The DASH score: a simple score to assess risk for development of malignant middle cerebral artery infarction. *Journal of the neurological sciences* **338,** 102–106 (2014).
11. Ong, C. J., Gluckstein, J., Laurido-Soto, O., *et al.* Enhanced Detection of Edema in Malignant Anterior Circulation Stroke (EDEMA) Score: A Risk Prediction Tool. *Stroke* **48,** 1969–1972 (2017).
12. Cheng, Y. *et al.* External validation and modification of the EDEMA score for predicting malignant brain edema after acute ischemic stroke. *Neurocritical care* **32,** 104–112 (2020).
13. Wu, S. *et al.* Predicting the emergence of malignant brain oedema in acute ischaemic stroke: a prospective multicentre study with development and validation of predictive modelling. *Eclinicalmedicine* **59** (2023).
14. Tang, A. *et al.* External validation and comparison of MBE, EDEMA, and modified EDEMA scores for predicting malignant cerebral EDEMA in Chinese patients with large hemisphereic infarction patients without revascularization. *Journal of Clinical Neuroscience* **122,** 66–72 (2024).

Supplementary Figures

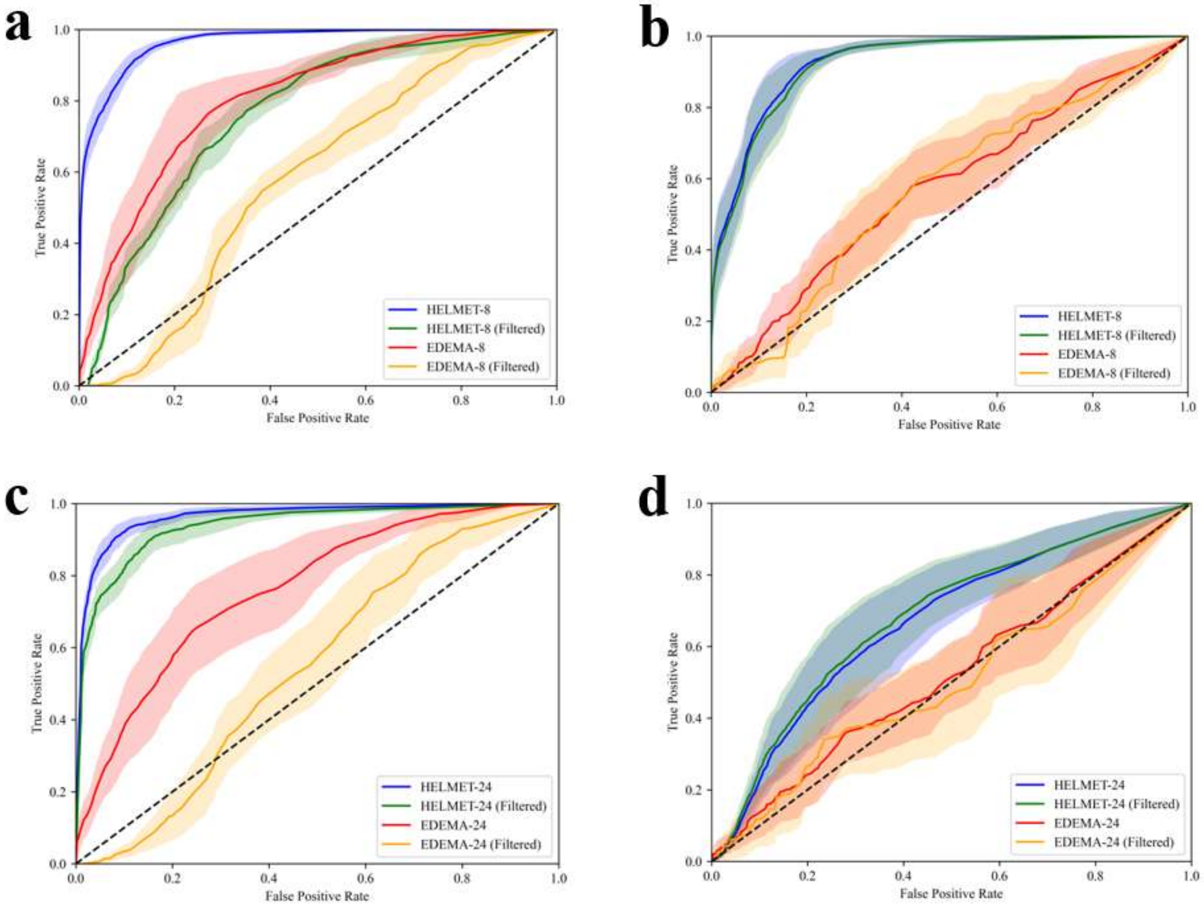

Supplementary Figure 1: **Receiver Operating Characteristic Curves.** Receiver operating characteristic curves for HELMET models compared to baseline EDEMA Score models for overall and filtered datasets for both derivation and external validation cohorts. Blue lines show performance of the HELMET models on the overall dataset, Orange lines show performance of the HELMET models on the filtered (MLS transition) observations. Red and orange lines show performance of the baseline EDEMA Score models on the overall and filtered datasets, respectively. a) Performance on the derivation dataset for the 8-hour prediction task; b) performance on external validation dataset for the 8-hour prediction task; c) derivation cohort performance for 24-hour task; d) external validation cohort performance for 24-hour task

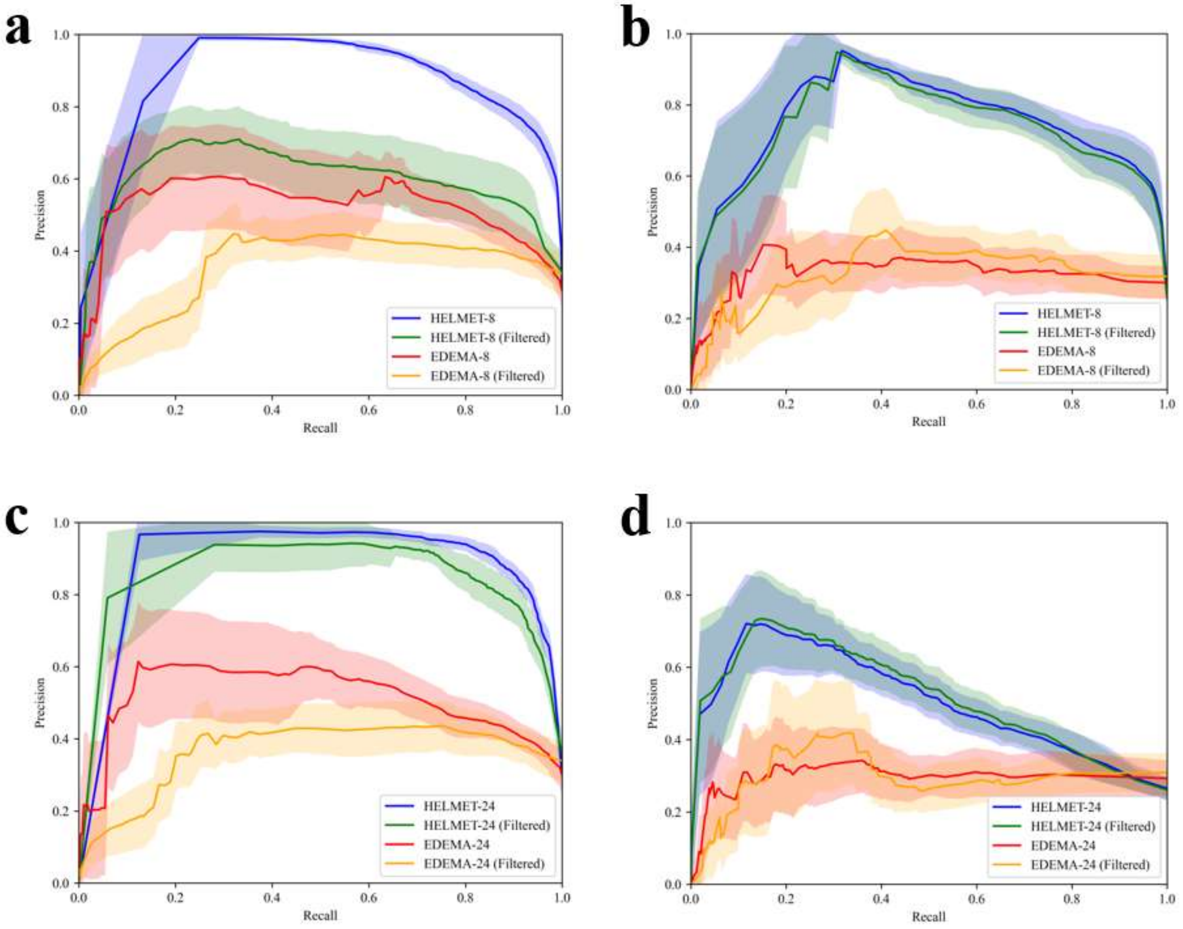

Supplementary Figure 2: **Precision Recall Curves.** Precision recall curves for HELMET models compared to baseline EDEMA Score models for overall and filtered datasets for both derivation and external validation cohorts. Blue lines show performance of the HELMET models on the overall dataset, Orange lines show performance of the HELMET models on the filtered (MLS transition) observations. Red and orange lines show performance of the baseline EDEMA Score models on the overall and filtered datasets, respectively. a) Performance on the derivation dataset for the 8-hour prediction task; b) performance on external validation dataset for the 8-hour prediction task; c) derivation cohort performance for 24-hour task; d) external validation cohort performance for 24-hour task

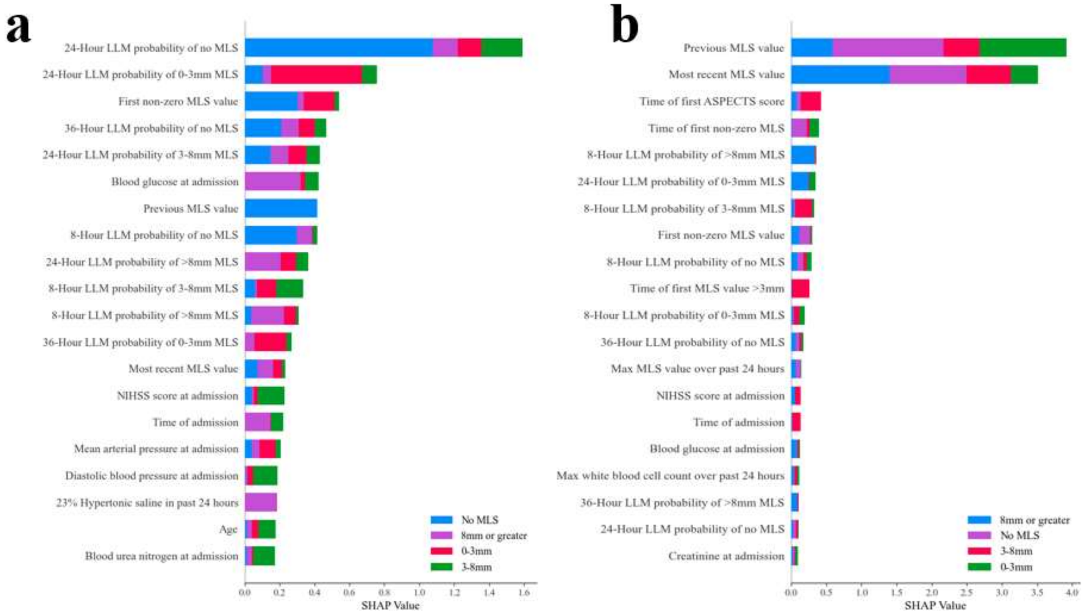

Supplementary Figure 3: **SHAP Plots.** The 20 features with the highest SHapley Additive exPlanation (SHAP) values for the ensemble learning models. Colors represent the contribution of each feature to positive predictions of each MLS class. a) Highest-ranked features for HELMET-24; b) Highest-ranked features for HELMET-8.

TRIPOD+AI

Version: 11-January-2024

Section/Topic	Item	Development / evaluation[1]	Checklist item	Reported on page
**TITLE**				
Title	1	D;E	Identify the study as developing or evaluating the performance of a multivariable prediction model, the target population, and the outcome to be predicted	1
**ABSTRACT**				
Abstract	2	D;E	See TRIPOD+AI for Abstracts checklist	
**INTRODUCTION**				
Background	3a	D;E	Explain the healthcare context (including whether diagnostic or prognostic) and rationale for developing or evaluating the prediction model, including references to existing models	2
	3b	D;E	Describe the target population and the intended purpose of the prediction model in the context of the care pathway, including its intended users (e.g., healthcare professionals, patients, public)	2-3
	3c	D;E	Describe any known health inequalities between sociodemographic groups	3
Objectives	4	D;E	Specify the study objectives, including whether the study describes the development or validation of a prediction model (or both)	3
**METHODS**				
Data	5a	D;E	Describe the sources of data separately for the development and evaluation datasets (e.g., randomised trial, cohort, routine care or registry data), the rationale for using these data, and representativeness of the data	8
	5b	D;E	Specify the dates of the collected participant data, including start and end of participant accrual; and, if applicable, end of follow-up	8
Participants	6a	D;E	Specify key elements of the study setting (e.g., primary care, secondary care, general population) including the number and location of centres	8
	6b	D;E	Describe the eligibility criteria for study participants	8 + Fig 1
	6c	D;E	Give details of any treatments received, and how they were handled during model development or evaluation, if relevant	9
Data preparation	7	D;E	Describe any data pre-processing and quality checking, including whether this was similar across relevant sociodemographic groups	9-10
Outcome	8a	D;E	Clearly define the outcome that is being predicted and the time horizon, including how and when assessed, the rationale for choosing this outcome, and whether the method of outcome assessment is consistent across sociodemographic groups	9
	8b	D;E	If outcome assessment requires subjective interpretation, describe the qualifications and demographic characteristics of the outcome assessors	9-10
	8c	D;E	Report any actions to blind assessment of the outcome to be predicted	NA
Predictors	9a	D	Describe the choice of initial predictors (e.g., literature, previous models, all available predictors) and any pre-selection of predictors before model building	11
	9b	D;E	Clearly define all predictors, including how and when they were measured (and any actions to blind assessment of predictors for the outcome and other predictors)	9-10
	9c	D;E	If predictor measurement requires subjective interpretation, describe the qualifications and demographic characteristics of the predictor assessors	9-10
Sample size	10	D;E	Explain how the study size was arrived at (separately for development and evaluation), and justify that the study size was sufficient to answer the research question. Include details of any sample size calculation	8
Missing data	11	D;E	Describe how missing data were handled. Provide reasons for omitting any data	9
Analytical methods	12a	D	Describe how the data were used (e.g., for development and evaluation of model performance) in the analysis, including whether the data were partitioned, considering any sample size requirements	10
	12b	D	Depending on the type of model, describe how predictors were handled in the analyses (functional form, rescaling, transformation, or any standardisation).	9-10
	12c	D	Specify the type of model, rationale[2], all model-building steps, including any hyperparameter tuning, and method for internal validation	10-11
	12d	D;E	Describe if and how any heterogeneity in estimates of model parameter values and model performance was handled and quantified across clusters (e.g., hospitals, countries). See TRIPOD-Cluster for additional considerations[3]	NA
	12e	D;E	Specify all measures and plots used (and their rationale) to evaluate model performance (e.g., discrimination, calibration, clinical utility) and, if relevant, to compare multiple models	11-12
	12f	E	Describe any model updating (e.g., recalibration) arising from the model evaluation, either overall or for particular sociodemographic groups or settings	NA
	12g	E	For model evaluation, describe how the model predictions were calculated (e.g., formula, code, object, application programming interface)	11
Class imbalance	13	D;E	If class imbalance methods were used, state why and how this was done, and any subsequent methods to recalibrate the model or the model predictions	NA
Fairness	14	D;E	Describe any approaches that were used to address model fairness and their rationale	NA
Model output	15	D	Specify the output of the prediction model (e.g., probabilities, classification). Provide details and rationale for any classification and how the thresholds were identified	9, 11

[1] D=items relevant only to the development of a prediction model; E=items relating solely to the evaluation of a prediction model; D;E=items applicable to both the development and evaluation of a prediction model

[2] Separately for all model building approaches.

[3] TRIPOD-Cluster is a checklist of reporting recommendations for studies developing or validating models that explicitly account for clustering or explore heterogeneity in model performance (eg, at different hospitals or centres). Debray et al, BMJ 2023; 380: e071018 [DOI: 10.1136/bmj-2022-071018]

Page 1 of 2

TRIPOD+AI

Version: 11-January-2024

Training versus evaluation	16	D;E	Identify any differences between the development and evaluation data in healthcare setting, eligibility criteria, outcome, and predictors	NA
Ethical approval	17	D;E	Name the institutional research board or ethics committee that approved the study and describe the participant-informed consent or the ethics committee waiver of informed consent	8
**OPEN SCIENCE**				
Funding	18a	D;E	Give the source of funding and the role of the funders for the present study	12
Conflicts of interest	18b	D;E	Declare any conflicts of interest and financial disclosures for all authors	13
Protocol	18c	D;E	Indicate where the study protocol can be accessed or state that a protocol was not prepared	NA
Registration	18d	D;E	Provide registration information for the study, including register name and registration number, or state that the study was not registered	NA
Data sharing	18e	D;E	Provide details of the availability of the study data	12
Code sharing	18f	D;E	Provide details of the availability of the analytical code[4]	12
**PATIENT & PUBLIC INVOLVEMENT**				
Patient & Public Involvement	19	D;E	Provide details of any patient and public involvement during the design, conduct, reporting, interpretation, or dissemination of the study or state no involvement.	NA
**RESULTS**				
Participants	20a	D;E	Describe the flow of participants through the study, including the number of participants with and without the outcome and, if applicable, a summary of the follow-up time. A diagram may be helpful.	3 + Fig 1
	20b	D;E	Report the characteristics overall and, where applicable, for each data source or setting, including the key dates, key predictors (including demographics), treatments received, sample size, number of outcome events, follow-up time, and amount of missing data. A table may be helpful. Report any differences across key demographic groups.	3 + Tab 1
	20c	E	For model evaluation, show a comparison with the development data of the distribution of important predictors (demographics, predictors, and outcome).	4-5 + Fig 4
Model development	21	D;E	Specify the number of participants and outcome events in each analysis (e.g., for model development, hyperparameter tuning, model evaluation)	3
Model specification	22	D	Provide details of the full prediction model (e.g., formula, code, object, application programming interface) to allow predictions in new individuals and to enable third-party evaluation and implementation, including any restrictions to access or re-use (e.g., freely available, proprietary)[5]	12
Model performance	23a	D;E	Report model performance estimates with confidence intervals, including for any key subgroups (e.g., sociodemographic). Consider plots to aid presentation.	4 + Fig 3
	23b	D;E	If examined, report results of any heterogeneity in model performance across clusters. See TRIPOD Cluster for additional details[3].	NA
Model updating	24	E	Report the results from any model updating, including the updated model and subsequent performance	NA
**DISCUSSION**				
Interpretation	25	D;E	Give an overall interpretation of the main results, including issues of fairness in the context of the objectives and previous studies	5-6
Limitations	26	D;E	Discuss any limitations of the study (such as a non-representative sample, sample size, overfitting, missing data) and their effects on any biases, statistical uncertainty, and generalizability	7
Usability of the model in the context of current care	27a	D	Describe how poor quality or unavailable input data (e.g., predictor values) should be assessed and handled when implementing the prediction model	6-7
	27b	D	Specify whether users will be required to interact in the handling of the input data or use of the model, and what level of expertise is required of users	6-7
	27c	D;E	Discuss any next steps for future research, with a specific view to applicability and generalizability of the model	5-7

From: Collins GS, Moons KGM, Dhiman P, et al. *BMJ* 2024;385:e078378. doi:10.1136/bmj-2023-078378

[4] This relates to the analysis code, for example, any data cleaning, feature engineering, model building, evaluation.
[5] This relates to the code to implement the model to get estimates of risk for a new individual.

Page 2 of 2

Supplementary Figure 4: **TRIPOD+AI Reporting Checklist.** Completed reporting guidelines checklist. Reporting follows the updated Transparent Reporting of a multivariable prediction model for Individual Prognosis Or Diagnosis guidelines (TRIPOD+AI).

Supplementary Tables

Supplementary Table 1: **Prior Literature.** A summary of existing research on predicting events relating to cerebral edema.

Study	Sample Size	Type	Prediction Target	AUROC	Dynamic Prediction?	Single Center?	Retrospective?	Other Limitations
Shimoyama et al., 2014 [10]	119	Logistic Regression	Malignant MCA infarction	0.88	No	Yes	Yes	Small sample biased towards an elderly population (average age 78.0)
Ong et al., 2017 [11]	222	Logistic Regression	Malignant brain edema	0.75	No	Yes	Yes	Late outcome prediction and only data from first 24 hours.
Cheng et al., 2020 [12]	487	Logistic Regression	Malignant brain edema	0.8	No	Yes	Yes	Same as Ong, et al., 2017. Also shows low generalizability without model re-training.
Wu et al., 2023 [13]	1627	Logistic Regression	Malignant brain edema	0.9	No	Yes	Yes	No imaging features used in the model.
Tang et al., 2024 [14]	314	Logistic Regression	Malignant brain edema	0.88	No	Yes	Yes	Homogeneous sample composition biased toward elderly population (average age 75.7)

Supplementary Table 2: **Static Variable Missingness.** Missingness in static variables across cohorts. All variables presented as patient counts of missing data (percentage of patients). Statistical significance of differences between cohorts tested using χ^2 test.

Variable	Massachusetts General Brigham	Boston Medical Center	p-value
**Demographic Factors**			
Age	0 (0%)	0 (0%)	1.000
Sex	0 (0%)	0 (0%)	1.000
Race	0 (0%)	0 (0%)	1.000
**Medical History**			
Previous stroke	0 (0%)	0 (0%)	1.000
Atrial fibrillation	0 (0%)	0 (0%)	1.000
Hypertension	0 (0%)	0 (0%)	1.000
**Stroke Characteristics at Admission**			
NIHSS	46 (7.4%)	0 (0%)	0.056
ASPECTS	0 (0%)	0 (0%)	1.000
Stroke side	0 (0%)	0 (0%)	1.000
Anterior cerebral artery involved	0 (0%)	0 (0%)	1.000
Vessel Occlusion	0 (0%)	0 (0%)	1.000
Collateral Score	207 (33.2%)	28 (46.7%)	0.051
First MLS	157 (25.2%)	0 (0%)	0.000
Cerebral Atrophy	3 (0.5%)	0 (0%)	1.000
**Vital Signs at Admission**			
Mean arterial pressure	43 (6.9%)	0 (0%)	0.068
Systolic blood pressure	0 (0%)	0 (0%)	1.000
Diastolic blood pressure	43 (6.9%)	0 (0%)	0.068
Heart rate	454 (72.9%)	0 (0%)	0.000
Body temperature	454 (72.9%)	0 (0%)	0.000
**Laboratory Values at Admission**			
White blood cell count	3 (0.5%)	0 (0%)	1.000
Blood glucose	2 (0.3%)	0 (0%)	1.000
HbA1c	98 (15.7%)	3 (5.0%)	0.041
Osmolality	445 (71.4%)	31 (51.7%)	0.002
Creatinine	3 (0.5%)	0 (0%)	1.000
Sodium	2 (0.3%)	0 (0%)	1.000
Blood urea nitrogen	3 (0.5%)	0 (0%)	1.000
**Treatments Administered**			
Medical Thrombolysis	0 (0%)	0 (0%)	1.000
Mechanical Thrombectomy	0 (0%)	0 (0%)	1.000
Decompressive Hemicraniectomy	0 (0%)	0 (0%)	1.000
Osmotic Therapies	0 (0%)	0 (0%)	1.000
**Clinical Outcomes**			
Modified Rankin Scale	121 (19.4%)	0 (0%)	0.000

Supplementary Table 3: **Dynamic Variable Updates.** Percent of patient-hour observations over the first week of hospitalization with an updated dynamic variable measurement in the past 8 hours.

Variable	Massachusetts General Brigham	Boston Medical Center	p-value
**Vital Signs**			
Heart rate	27.3%	98.0%	0.000
Systolic blood pressure	18.8%	96.4%	0.000
Diastolic blood pressure	18.8%	96.4%	0.000
Body temperature	27.8%	96.1%	0.000
**Laboratory Values**			
White blood cell count	45.5%	43.3%	0.657
Blood glucose	69.9%	71.3%	0.757
Osmolality	18.9%	20.3%	0.728
Creatinine	58.1%	49.0%	0.069
Sodium	63.9%	68.1%	0.368
Blood urea nitrogen	58.1%	49.1%	0.072
**Radiographic Measurements**			
Midline shift	25.6%	27.5%	0.670
Pineal gland shift	25.6%	27.1%	0.736

Supplementary Table 4: **Dynamic Variable Frequency.** Average time delay between updated measurements in dynamic variables within the first week of hospitalization across cohorts. All variables presented as mean hours (SD). Statistical significance of differences between cohorts tested using Mann-Whitney U test.

Variable	Massachusetts General Brigham	Boston Medical Center	p-value
**Vital Signs**			
Heart rate	6.1 (4.1)	3.1 (4.2)	0.000
Systolic blood pressure	7.1 (4.3)	2.6 (3.8)	0.000
Diastolic blood pressure	7.6 (4.7)	3.1 (3.9)	0.000
Body temperature	8.8 (6.5)	5.9 (7.0)	0.000
**Laboratory Values**			
White blood cell count	8.6 (7.2)	13.8 (17.8)	0.003
Blood glucose	6.0 (5.3)	6.8 (7.6)	0.652
Osmolality	11.8 (8.2)	21.1 (23.5)	0.008
Creatinine	7.8 (6.3)	12.3 (12.8)	0.002
Sodium	7.7 (6.2)	10.2 (11.6)	0.286
Blood urea nitrogen	8.2 (6.3)	13.2 (15.6)	0.003
**Radiographic Measurements**			
Midline shift	11.3 (9.1)	18.8 (22.7)	0.003
Pineal gland shift	11.6 (9.1)	18.9 (22.4)	0.008

Supplementary Table 5: **Edema Severity.** Maximum MLS class reached by patients across cohorts and prediction tasks.

Data set	Task	Maximum MLS class			
		0mm	0-3mm	3-8mm	>8mm
Mass General Brigham	8-hour	162	100	216	145
(derivation cohort)	24-hour	123	81	204	125
Boston Medical Center	8-hour	10	10	17	23
(validation cohort)	24-hour	9	9	18	19

Supplementary Table 6: **Hospitalization Characteristics.** Average characteristics of patient hospitalization by initial MLS class using full 8-hour task cohorts.

**Data set**	**Characteristic**	**Initial MLS class**			
		**0mm**	**0-3mm**	**3-8mm**	**>8mm**
Mass General Brigham (derivation cohort)	Number of Patients, count (%)	535 (85.9%)	48 (7.7%)	40 (6.4%)	*None*
	Length of Stay (days), median (IQR)	2.2 (4.0)	1.4 (3.2)	3.1 (4.8)	*None*
	Hours Between Scans*, mean (SD)	21.6 (13.0)	21.5 (14.2)	28.8 (31.6)	*None*
	Maximum MLS (mm), mean (SD)	3.3 (4.2)	4.4 (3.6)	7.2 (3.7)	*None*
Boston Medical Center (validation cohort)	Number of Patients, count (%)	49 (81.7%)	9 (15.0%)	2 (3.3%)	*None*
	Length of Stay (days), median (IQR)	3.9 (4.9)	5.2 (3.8)	3.4 (2.4)	*None*
	Hours Between Scans*, mean (SD)	21.8 (9.2)	25.4 (11.4)	26.6 (11.8)	*None*
	Maximum MLS (mm), mean (SD)	5.1 (5.2)	7.9 (4.9)	10.2 (5.9)	*None*

**Frequency of scanning calculated using first 7 days of hospitalization due to infrequent scanning in later periods*

Supplementary Table 7: **Complete list of features used by HELMET-24.**

Feature	Relative Importance	Description	Source
LLM_24_3	53.3	LLM-assessed probability of transition to class 3 over next 24 hours	Radiology Report Texts
LLM_24_2	46.8	LLM-assessed probability of transition to class 2 over next 24 hours	Radiology Report Texts
LLM_24_1	41.4	LLM-assessed probability of transition to class 1 over next 24 hours	Radiology Report Texts
LLM_24_0	29.1	LLM-assessed probability of transition to class 0 over next 24 hours	Radiology Report Texts
LLM_36_1	11.5	LLM-assessed probability of transition to class 1 over next 36 hours	Radiology Report Texts
wbc1	11.0	White blood cell count at admission	Patient Medical Record
LLM_8_0	8.0	LLM-assessed probability of transition to class 0 over next 8 hours	Radiology Report Texts
rolling_map	7.8	Maximum mean arterial pressure over past 24 hours	Patient Medical Record
LLM_8_2	7.5	LLM-assessed probability of transition to class 2 over next 8 hours	Radiology Report Texts
firstmls_time_censored	7.4	Value of first measured MLS	Radiographic Images
rolling_pulse	7.3	Maximum pulse over past 24 hours	Patient Medical Record
LLM_36_0	6.9	LLM-assessed probability of transition to class 0 over next 36 hours	Radiology Report Texts
mls5dt_time_censored	6.8	Time at which patient first reached MLS of 5mm or greater	Radiographic Images
temp	6.7	Most recent body temperature	Patient Medical Record
prev_mls	6.5	Last measured MLS value prior to current value	Radiographic Images
LLM_36_2	6.4	LLM-assessed probability of transition to class 2 over next 36 hours	Radiology Report Texts
LLM_8_3	5.9	LLM-assessed probability of transition to class 3 over next 8 hours	Radiology Report Texts
gluc1	5.7	Blood glucose at admission	Patient Medical Record
hi2	5.6	Petechial hemorrhage gaining confluence in most recent scan	Radiographic Images
rolling_size_mls	5.6	Maximum MLS value over past 24 hours	Radiographic Images
pulse1	5.1	Pulse at admission	Patient Medical Record
size_mls	4.9	Most recent MLS value	Radiographic Images
rolling_temp	4.8	Maximum body temperature over past 24 hours	Patient Medical Record
pgs2_time_censored	4.7	Value of maximum pineal gland shift of 2mm or greater so far	Radiographic Images
pgs4dt_time_censored	4.7	Time at which pineal gland shift first reached 4mm or greater	Radiographic Images
temp1	4.7	Body temperature at admission	Patient Medical Record
hts23_x	4.5	Administration of hypertonic saline (concentration 23.4%)	Patient Medical Record
evd	4.5	Extraventricular drain in most recent scan	Radiographic Images
aspects	4.4	Most recent ASPECTS index score	Radiographic Images
rolling_hts3_y	4.1	Whether hypertonic saline (concentration 3%) has been administered	Patient Medical Record
aspects1dt_time_censored	4.1	Time of first ASPECTS index score	Radiographic Images
pres	4.0	Time of admission	Patient Medical Record
map1	4.0	Mean arterial pressure at admission	Patient Medical Record
LLM_36_3	4.0	LLM-assessed probability of transition to class 3 over next 36 hours	Radiology Report Texts
bun1	4.0	Blood urea nitrogen at admission	Patient Medical Record
wbc	4.0	Most recent white blood cell count	Patient Medical Record
imagetype	3.8	Categorical representation of most recent scan type	Radiographic Images
rolling_aca_x	3.7	Whether anterior cerebral artery has been involved in past 24 hours	Radiographic Images

Continued on next page

Supplementary Table 7 – continued from previous page

Feature	Relative Importance	Description Source	
rolling_hts23_y	3.7	Whether hypertonic saline (concentration 23.4%) has been administered	Patient Medical Record
firstmlsdt_time_censored	3.5	Time of first MLS measurement greater than 0	Radiographic Images
stroke_territory	3.5	Size of affected stroke territory	Radiographic Images
rolling_osm	3.5	Maximum osmolality value over past 24 hours	Patient Medical Record
mls3dt_time_censored	3.5	Time of first MLS measurement of 3mm or greater	Radiographic Images
sbp1	3.5	Systolic blood pressure at admission	Patient Medical Record
rolling_size_pgs	3.4	Maximum pineal gland shift over past 24 hours	Radiographic Images
rolling_gluc	3.3	Maximum blood glucose over past 24 hours	Patient Medical Record
rolling_sbp	3.3	Maximum systolic blood pressure over past 24 hours	Patient Medical Record
sbp	3.3	Most recent systolic blood pressure	Patient Medical Record
age_calc	3.3	Age	Patient Medical Record
cr	3.3	Most recent creatinine value	Patient Medical Record
dbp1	3.2	Diastolic blood pressure at admission	Patient Medical Record
rolling_dbp	3.2	Maximum diastolic blood pressure over past 24 hours	Patient Medical Record
rolling_wbc	3.1	Maximum white blood cell count over past 24 hours	Patient Medical Record
LLM_8_1	3.1	LLM-assessed probability of transition to class 1 over next 8 hours	Radiology Report Texts
aspects1_time_censored	3.0	ASPECTS score from first scan	Radiographic Images
osm	3.0	Most recent osmolality value	Patient Medical Record
mtdt_time_censored	3.0	Time of mechanical thrombectomy	Patient Medical Record
aca_x	2.9	Whether anterior cerebral artery is involved in most recent scan	Radiographic Images
htn	2.9	History of hypertension	Patient Medical Record
rolling_mannitol_y	2.9	Whether mannitol has been administered	Patient Medical Record
stroke	2.9	Occurrence of previous stroke	Patient Medical Record
rolling_na	2.8	Maximum sodium over past 24 hours	Patient Medical Record
dbp	2.8	Most recent diastolic blood pressure value	Patient Medical Record
af	2.7	History of atrial fibrillation	Patient Medical Record
na1	2.7	Sodium at admission	Patient Medical Record
mt_time_censored	2.7	Whether mechanical thrombectomy has occurred	Patient Medical Record
dt	2.7	Hours since Last Seen Well	Patient Medical Record
rolling_bun	2.6	Maximum blood urea nitrogen in past 24 hours	Patient Medical Record
rolling_imagetype	2.6	Most intensive scan type in last 24 hours	Radiographic Images
rolling_aspects	2.6	Maximum ASPECTS score in past 24 hours	Radiographic Images
bun	2.6	Most recent blood urea nitrogen value	Patient Medical Record
nihss	2.5	NIHSS score at admission	Patient Medical Record
sex	2.5	Female or not	Patient Medical Record
tpa_time_censored	2.5	Administration of TPA	Patient Medical Record
rolling_hts3_x	2.4	Administration of hypertonic saline (concentration 3%) in past 24 hours	Patient Medical Record
size_pgs	2.4	Most recent pineal gland shift measurement	Radiographic Images

Continued on next page

Supplementary Table 7 – continued from previous page

Feature	Relative Importance	Description	Source
hts3_x	2.4	Administration of hypertonic saline (concentration 3%) in current hour	Patient Medical Record
tpadt_time_censored	2.4	Time at which TPA was administered	Patient Medical Record
rolling_cr	2.4	Maximum creatinine value in past 24 hours	Patient Medical Record
rolling_osmotics_y	2.4	Whether osmotic treatment has been administered	Patient Medical Record
mls12dt_time_censored	2.4	Time at which MLS first reached 12mm or greater	Radiographic Images
mannitol_x	2.3	Administration of mannitol in current hour	Patient Medical Record
gluc	2.3	Most recent blood glucose value	Patient Medical Record
na	2.3	Most recent sodium value	Patient Medical Record
rolling_hts23_x	2.2	Administration of hypertonic saline (concentration 23.4%) in past 24 hours	Patient Medical Record
obsTime	2.2	Current date and time	
cr1	2.2	Creatinine at admission	Patient Medical Record
rolling_hi1	2.1	Petechial hemorrhage in past 24 hours	Radiographic Images
rolling_stroke_territory	2.0	Maximum size of stroke territory affected in last 24 hours	Radiographic Images
rolling_bce_severity	2.0	Maximum basilar cistern effacement in past 24 hours	Radiographic Images
pgs2dt_time_censored	1.9	Time at which pineal gland shift first reached 2mm or greater	Radiographic Images
rolling_hi2	1.9	Petechial hemorrhages starting to gain confluence in past 24 hours	Radiographic Images
map	1.9	Most recent mean arterial pressure value	Patient Medical Record
hi1	1.8	Petechial hemorrhage present in most recent scan	Radiographic Images
rolling_aca_y	1.8	Whether anterior cerebral artery was ever involved	Radiographic Images
rolling_evd	1.6	Extraventricular drain in past 24 hours	Radiographic Images
pulse	1.6	Most recent pulse	Patient Medical Record
rolling_mannitol_x	1.5	Administration of mannitol in past 24 hours	Patient Medical Record

Supplementary Table 8: **Complete list of features used by HELMET-8.**

Feature	Relative Importance	Description	Source
prev_mls	72.7	Last measured MLS value prior to current value	
LLM_8_2	48.9	LLM-assessed probability of transition to class 2 over next 8 hours	
size_mls	47.1	Most recent MLS measurement	
LLM_8_3	42.0	LLM-assessed probability of transition to class 3 over next 8 hours	
LLM_8_1	37.4	LLM-assessed probability of transition to class 1 over next 8 hours	
firstmlsdt_time_censored	14.0	Time of first non-zero MLS measurement	
mls3dt_time_censored	13.6	Time of first MLS measurement greater than 3mm	
LLM_8_0	12.5	LLM-assessed probability of transition to class 0 over next 8 hours	
firstmls_time_censored	10.7	Value of first non-zero MLS measurement	
pulse1	9.6	Pulse at admission	
temp1	9.4	Body temperature at admission	
rolling_hts3_x	8.5	Whether hypertonic saline (concentration 3%) has been administered in past 24 hours	
LLM_24_3	8.2	LLM-assessed probability of transition to class 3 over next 24 hours	
LLM_24_1	7.8	LLM-assessed probability of transition to class 1 over next 24 hours	
dbp	7.5	Most recent diastolic blood pressure	
rolling_temp	7.1	Maximum body temperature in past 24 hours	
LLM_24_0	6.9	LLM-assessed probability of transition to class 0 over next 24 hours	
pgs2dt_time_censored	6.8	Time of first pineal gland shift measurement greater than 2mm	
LLM_36_3	6.5	LLM-assessed probability of transition to class 3 over next 36 hours	
aspects1dt_time_censored	6.3	Time of first ASPECTS score	
pulse	6.0	Most recent pulse	
LLM_24_2	5.9	LLM-assessed probability of transition to class 2 over next 24 hours	
imagetype	5.9	Category of most recent scan	
pres	5.9	Time of admission	
LLM_36_0	5.7	LLM-assessed probability of transition to class 0 over next 36 hours	
rolling_dbp	5.6	Maximum diastolic blood pressure in past 24 hours	
LLM_36_2	5.5	LLM-assessed probability of transition to class 2 over next 36 hours	
rolling_imagetype	5.4	Most intense scan in past 24 hours	
af	5.4	History of atrial fibrillation	
LLM_36_1	5.3	LLM-assessed probability of transition to class 1 over next 36 hours	
mt_time_censored	5.3	Whether mechanical thrombectomy has occurred	
pgs4dt_time_censored	5.1	Time of first pineal gland shift measurement greater than 4mm	
rolling_osmotics_y	5.1	Whether osmotics have been administered	
rolling_sbp	4.9	Maximum systolic blood pressure in past 24 hours	
hts23_x	4.8	Administration of hypertonic saline (concentration 23.4%) in current hour	
tpa_time_censored	4.6	Whether TPA has been administered	
rolling_aspects	4.6	Maximum ASPECTS score in past 24 hours	
stroke_territory	4.5	Size of affected stroke territory on most recent scan	

Continued on next page

Supplementary Table 8 – continued from previous page

Feature	Relative Importance	Description	Source
rolling_aca_x	4.4	Whether anterior cerebral artery has been affected in past 24 hours	
cr1	4.4	Creatine at admission	
wbc	4.4	Most recent white blood cell count	
rolling_hts23_x	4.4	Whether hypertonic saline (concentration 23.4%) has been administered in past 24 hours	
rolling_map	4.3	Maximum mean arterial pressure in past 24 hours	
rolling_pulse	4.2	Maximum pulse in past 24 hours	
rolling_size_pgs	4.1	Maximum pineal gland shift measurement in past 24 hours	
rolling_size_mls	4.1	Maximum MLS measurement in past 24 hours	
wbc1	4.1	White blood cell count at admission	
aspects1_time_censored	4.1	ASPECTS score at admission	
bun	4.1	Most recent blood urea nitrogen value	
dbp1	4.1	Diastolic blood pressure at admission	
size_pgs	4.0	Most recent pineal gland shift measurement	
rolling_wbc	4.0	Maximum white blood cell count in past 24 hours	
mtdt_time_censored	4.0	Time of mechanical thrombectomy	
cr	4.0	Most recent creatinine value	
na	4.0	Most recent sodium value	
obsTime	3.9	Current time	
map1	3.9	Mean arterial pressure at admission	
aca_x	3.9	Whether anterior cerebral artery was involved in most recent scan	
rolling_stroke_territory	3.8	Maximum size of affected stroke territory in past 24 hours	
rolling_aca_y	3.8	Whether anterior cerebral artery has been involved in scans in the past 24 hours	
rolling_gluc	3.8	Maximum blood glucose in past 24 hours	
gluc1	3.7	Blood glucose at admission	
temp	3.7	Most recent body temperature	
map	3.7	Most recent mean arterial pressure	
tpadt_time_censored	3.7	Time at which TPA was administered	
bun1	3.6	Blood urea nitrogen at admission	
aca1	3.5	Whether anterior cerebral artery was involved at first scan	
rolling_mannitol_x	3.5	Whether mannitol has been administered in last 24 hours	
nihss	3.5	NIHSS score at admission	
age_calc	3.5	Age	
na1	3.4	Sodium at admission	
sbp	3.4	Most recent systolic blood pressure	
dt	3.4	Current time	
rolling_mannitol_y	3.4	Whether mannitol has been administered at any point	
rolling_cr	3.2	Maximum creatinine value in past 24 hours	
mannitol_x	3.1	Administration of mannitol	

Continued on next page

Supplementary Table 8 – continued from previous page

Feature	Relative Importance	Description	Source
sbp1	3.1	Systolic blood pressure at admission	
aspects	3.0	Most recent ASPECTS score	
rolling_na	2.9	Maximum sodium value in past 24 hours	
sex	2.8	Female or not	
osm	2.6	Most recent osmolality value	
gluc	2.6	Most recent blood glucose value	
rolling_bce_severity	2.5	Maximum basilar cistern effacement in past 24 hours	
rolling_osm	2.4	Maximum osmolality in past 24 hours	
rolling_bun	2.4	Maximum blood urea nitrogen in past 24 hours	
rolling_hts3_y	2.3	Whether hypertonic saline (concentration 3%) has been administered	
hi2	2.2	Petechial hemorrhage on most recent scan	
mls7_time_censored	2.0	Value of maximum MLS measurement greater than 7mm	
mls7dt_time_censored	1.8	Time of first MLS measurement greater than 7mm	
pgs4_time_censored	1.8	Value of maximum pineal gland shift greater than 4mm	
mls5dt_time_censored	1.7	Time of first MLS measurement greater than 5mm	
rolling_hts23_y	1.6	Whether hypertonic saline (concentration 23.4%) has been administered	
hts3_x	1.6	Administration of hypertonic saline (concentration 3%)	
rolling_hi1	1.1	Petechial hemorrhage in past 24 hours	
rolling_hi2	0.1	Petechial hemorrhage gaining confluence in past 24 hours	

Supplementary Table 9: **Algorithm Comparison.** A comparison of XGBoost and Random Forest performance on five-fold splits on the derivation dataset.

Prediction Task	Model	AUROC	AUPRC	Accuracy
24 hours	Random Forrest	86.0%(84.51%, 87.49%)	68.0%(64.41%, 71.59%)	62.0%(58.32%, 65.68%)
	XGBoost	86.1%(85.37%, 86.83%)	68.7%(67.87%, 69.53%)	62.1%(60.53%, 63.67%)
8 hours	Random Forrest	86.0%(85.42%, 86.58%)	67.0%(65.33%, 68.67%)	63.0%(59.14%, 66.86%)
	XGBoost	86.2%(85.68%, 86.72%)	67.3%(66.84%, 67.76%)	62.8%(61.61%, 63.99%)

Supplementary Table 10: **Model Training Parameters.** Final hyperparameters used in training HELMET-24 and HELMET-8. Optimal hyperparameters were determined through cross-validation and bayesian optimizaiton across successive model training runs.

Parameter	HELMET-24	HELMET-8
Maximum Tree Depth	7	4
Minimum Child Weight	1	10
Learning Rate	0.0972	0.0669
Gamma	1.32	0.0831
Data Subsample	0.704	0.768
Regularization Lambda	6.07	6.62
Regularization Alpha	2.04	1.36
No-transition Sample Weight	0.475	0.563

Supplementary Table 11: **Complete Performance Metrics.** Comparison of performance metrics for HELMET and EDEMA models across derivation and external validation cohorts. Reported values were averaged across five cross-validation folds. The HELMET models were derived using the training set of the derivation cohort and were evaluated on the testing set of the derivation cohort and the entire external validation cohort. The EDEMA Regression Baseline models were separately trained and tested on both the derivation and external validation data. Values in parentheses reflect the 95% confidence intervals.

**Cohort**	**Metric**	**Type**	**24 hours ahead**		**8 hours ahead**	
			**EDEMA Baseline**	**HELMET-24**	**EDEMA Baseline**	**HELMET-8**
**Derivation Cohort**	AUROC	Overall	78.0% (71.3%, 84.7%)	96.7% (95.2%, 98.1%)	80.5% (74.1%, 86.9%)	96.6% (95.6%, 97.7%)
		Filtered	54.7% (41.7%, 67.6%)	94.1% (92.3%, 96.0%)	57.5% (46.3%, 68.7%)	76.2% (66.6%, 85.8%)
	AUPRC	Overall	57.6% (42,4%, 72.8%)	87.2% (83.2%, 91.2%)	59.3% (44.9%, 73.6%)	87.5% (82.9%, 92.1%)
		Filtered	37.1% (28.6%, 45.5%)	85.2% (78.1%, 92.2%)	37.3% (29.8%, 44.7%)	61.2% (46.9%, 75.5%)
	Accuracy	Overall	59.0% (53.5%, 64.4%)	87.3% (84.1%, 90.4%)	61.6% (57.8%, 65.3%)	82.9% (76.2%, 89.6%)
		Filtered	25.1% (18.1%, 32.0%)	81.2% (76.5%, 85.9%)	23.5% (18.1%, 28.8%)	46.7% (38.1%, 55.3%)
	Sensitivity	Overall	37.6% (30.4%, 44.8%)	91.2% (85.4%, 97.0%)	32.1% (26.2%, 37.9%)	57.7% (47.4%, 68.1%)
		Filtered	37.6% (30.4%, 44.8%)	91.2% (85.4%, 97.0%)	32.1% (26.2%, 37.9%)	57.7% (47.4%, 68.1%)
	Specificity	Overall	84.3% (78.0%, 90.6%)	94.0% (88.5%, 99.5%)	87.6% (77.8%, 97.4%)	90.2% (81.1%, 99.2%)
		Filtered	86.9% (77.0%, 96.9%)	97.8% (91.1%, 100.0%)	85.1% (64.3%, 100.0%)	75.6% (9.5%, 100.0%)
**External Validation Cohort**	AUROC	Overall	51.3% (42.7%, 59.9%)	69.7% (60.3%, 79.0%)	58.5% (48.5%, 68.4%)	92.5% (89.4%, 95.6%)
		Filtered	51.1% (40.8%, 61.4%)	70.7% (61.1%, 80.3%)	58.8% (48.7%, 68.8%)	92.1% (88.5%, 95.7%)
	AUPRC	Overall	33.0% (19.1%, 46.9%)	46.9% (35.6%, 58.1%)	38.9% (26.2%, 51.6%)	80.5% (69.8%, 91.3%)
		Filtered	35.3% (21.3%, 49.2%)	48.4% (36.2%, 60.6%)	40.2% (27.5%, 52.9%)	79.8% (70.3%, 89.3%)
	Accuracy	Overall	29.2% (16.7%, 41.6%)	48.6% (46.7%, 50.6%)	37.9% (5.3%, 70.5%)	75.3% (70.3%, 80.3%)
		Filtered	30.4% (22.5%, 38.3%)	48.7% (45.6%, 51.8%)	28.9% (7.8%, 49.9%)	73.3% (68.0%, 78.6%)
	Sensitivity	Overall	63.3% (37.3%, 89.3%)	87.4% (80.9%, 94.0%)	47.9% (17.8%, 78.1%)	92.1% (89.4%, 94.9%)
		Filtered	63.3% (37.3%, 89.3%)	87.4% (80.9%, 94.0%)	47.9% (17.8%, 78.1%)	92.1% (89.4%, 94.9%)
	Specificity	Overall	62.3% (44.8%, 79.7%)	80.6% (75.3%, 85.9%)	78.2% (52.3%, 100.0%)	94.1% (89.6%, 98.6%)
		Filtered	79.6% (63.8%, 95.3%)	95.0% (91.5%, 98.5%)	100.0% (100.0%, 100.0%)	99.2% (96.8%, 100.0%)

Supplementary Table 12: **Performance by Hospitalization Period.** Comparison of AUROC performance for HELMET and EDEMA models across derivation and external validation cohorts by period of hospitalization (hours since last seen well). Periods were determined based on clinician-identified timings of peak edema severity. Values in parentheses reflect the 95% confidence intervals. We report the proportion of observations in each hospitalization period based on the HELMET-8 testing data; observation counts may vary slightly for HELMET-24 and EDEMA-8/24 testing sets due to missing data.

Cohort	Hours after LSW	Type	AUROC for 24-hour predictions		AUROC for 8-hour predictions	
			EDEMA Baseline	HELMET-24	EDEMA Baseline	HELMET-8
Derivation Cohort	<24hrs (18.9% of observations)	Overall	64.1% (57.4%, 70.9%)	97.2% (95.8%, 98.6%)	67.6% (61.7%, 73.4%)	90.8% (87.9%, 93.7%)
		Filtered	54.4% (46.3%, 62.4%)	94.8% (93.3%, 96.3%)	62.5% (52.1%, 72.8%)	75.3% (65.8%, 84.8%)
	24-48hrs (28.6% of observations)	Overall	76.4% (69.1%, 83.8%)	96.7% (95.1%, 98.2%)	78.5% (71.6%, 85.3%)	94.5% (92.4%, 96.7%)
		Filtered	57.8% (43.5%, 72.2%)	94.5% (92.6%, 96.4%)	56.9% (44.2%, 69.5%)	71.3% (61.7%, 81.0%)
	48-96hrs (29.2% of observations)	Overall	80.6% (73.4%, 87.7%)	96.1% (94.1%, 98.1%)	80.7% (75.5%, 86.0%)	97.8% (96.6%, 99.1%)
		Filtered	57.4% (41.8%, 72.9%)	91.5% (88.0%, 95.1%)	56.5% (40.9%, 72.2%)	82.1% (67.2%, 97.0%)
	>96hrs (23.2% of observations)	Overall	81.1% (72.2%, 90.1%)	96.9% (94.4%, 99.3%)	83.8% (76.5%, 91.1%)	98.8% (98.2%, 99.4%)
		Filtered	52.9% (33.0%, 72.7%)	93.0% (90.2%, 95.8%)	51.2% (33.9%, 68.5%)	80.4% (64.8%, 95.9%)
External Validation Cohort	<24hrs (15.4% of observations)	Overall	52.8% (42.6%, 63.1%)	67.4% (56.3%, 78.6%)	62.5% (53.2%, 71.8%)	91.9% (87.4%, 96.4%)
		Filtered	48.8% (27.5%, 70.0%)	68.4% (55.5%, 81.3%)	64.7% (48.9%, 80.6%)	89.8% (85.0%, 94.7%)
	24-48hrs (23.9% of observations)	Overall	43.7% (35.5%, 51.8%)	68.0% (59.6%, 76.4%)	54.9% (38.8%, 70.9%)	92.1% (88.7%, 95.6%)
		Filtered	44.4% (32.2%, 56.5%)	67.6% (58.6%, 76.6%)	55.5% (32.8%, 78.3%)	90.4% (85.5%, 95.2%)
	48-96hrs (27.7% of observations)	Overall	53.9% (38.9%, 68.9%)	71.3% (60.9%, 81.6%)	53.0% (34.6%, 71.3%)	94.6% (91.3%, 97.9%)
		Filtered	55.5% (37.7%, 73.3%)	72.0% (62.7%, 81.3%)	57.8% (26.2%, 89.4%)	94.7% (91.2%, 98.2%)
	>96hrs (32.4% of observations)	Overall	44.7% (26.4%, 63.1%)	71.7% (60.6%, 82.9%)	65.1% (50.5%, 79.7%)	91.3% (87.7%, 95.0%)
		Filtered	49.0% (37.2%, 60.7%)	73.8% (62.2%, 85.4%)	59.5% (31.7%, 87.2%)	91.5% (86.9%, 96.1%)

Supplementary Table 13: **Performance by Insurance Status.** Comparison of AUROC performance for HELMET and EDEMA models by patient insurance status. Values in parentheses reflect the 95% confidence intervals. We report the number of patients in each insurance type subset calculated based on HELMET-8 testing data; patient counts may vary slightly for HELMET-24 and EDEMA-8/24 testing sets due to missing data. NA indicates that the metric could not be computed due to an insufficient number of observations in the data subset.

Cohort	Insurance Type	Metric Type	AUROC for 24-hour predictions		AUROC for 8-hour predictions	
			EDEMA Baseline	HELMET-24	EDEMA Baseline	HELMET-8
Derivation Cohort	Medicare/Private (n=381)	Overall	78.5% (71.5%, 85.5%)	96.3% (94.6%, 98.0%)	80.9% (74.2%, 87.5%)	96.7% (96.8%, 97.6%)
		Filtered	55.2% (42.7%, 67.8%)	93.3% (91.3%, 95.3%)	58.6% (48.0%, 69.4%)	75.7% (68.3%, 83.1%)
	Medicaid (n=124)	Overall	77.0% (69.7%, 84.4%)	97.5% (95.9%, 99.1%)	78.6% (71.6%, 85.6%)	96.2% (94.5%, 97.9%)
		Filtered	51.1% (35.2%, 67.0%)	94.7% (90.6%, 98.8%)	52.7% (37.1%, 68.2%)	75.6% (56.5%, 97.9%)
	Uninsured (n=20)	Overall	86.5% (74.1%, 98.9%)	98.7% (96.7%, 100.0%)	58.1% (44.1%, 72.2%)	78.3% (76.3%, 80.2%)
		Filtered	50.3% (31.0%, 69.6%)	74.8% (70.2%, 79.4%)	NA	NA
External Validation Cohort	Medicare/Private (n=25)	Overall	45.2% (32.0%, 58.4%)	66.3% (56.3%, 76.4%)	54.7% (42.0%, 67.3%)	92.5% (89.2%, 95.8%)
		Filtered	41.8% (20.5%, 63.2%)	65.9% (56.1%, 75.8%)	55.1% (34.0%, 76.2%)	91.9% (88.3%, 95.4%)
	Medicaid (n=27)	Overall	57.1% (46.2%, 68.0%)	71.0% (61.5%, 80.5%)	61.1% (50.2%, 72.0%)	92.5% (89.1%, 95.9%)
		Filtered	55.6% (45.0%, 66.2%)	73.2% (63.6%, 82.8%)	54.9% (39.4%, 70.5%)	92.4% (88.4%, 96.4%)
	Uninsured (n=3)	Overall	NA	80.4% (68.6%, 92.2%)	NA	92.7% (84.7%, 100.0%)
		Filtered	NA	70.7% (63.7%, 77.6%)	NA	90.2% (82.8%, 97.6%)

Supplementary Table 14: **Binary Task Performance.** Comparison of HELMET and EDEMA models for simplified binary classification task at MLS threshold of 5mm. All values shown as mean (SD) of 5-fold cross-validation testing. HELMET models were trained on derivation cohort data and evaluated on both cohorts, while EDEMA models were trained and evaluated on each cohort independently.

**Cohort**	**Metric**	**Type**	**24 hours ahead**		**8 hours ahead**	
			**EDEMA Baseline**	**HELMET-24**	**EDEMA Baseline**	**HELMET-8**
Derivation Cohort	AUROC	Overall	85.6% (3.1%)	88.4% (2.2%)	87.0% (3.9%)	89.1% (1.9%)
		Filtered	4.5% (3.5%)	61.7% (13.4%)	0.1% (0.3%)	32.6% (8.0%)
	AUPRC	Overall	85.2% (3.0%)	87.9% (2.1%)	87.8% (3.2%)	89.3% (2.2%)
		Filtered	60.0% (12.1%)	76.4% (5.9%)	53.6% (8.9%)	64.0% (8.5%)
	Accuracy	Overall	79.0% (4.3%)	80.4% (2.2%)	84.0% (3.7%)	84.5% (2.4%)
		Filtered	27.0% (9.2%)	49.6% (17.9%)	20.6% (12.6%)	54.5% (8.7%)
External Validation Cohort	AUROC	Overall	63.5% (32.6%)	91.0% (0.5%)	75.7% (38.1%)	89.7% (0.7%)
		Filtered	0.8% (1.6%)	55.6% (10.9%)	1.7% (3.3%)	26.0% (7.5%)
	AUPRC	Overall	86.0% (10.3%)	91.1% (0.7%)	96.0% (4.7%)	90.0% (0.7%)
		Filtered	64.4% (19.2%)	69.4% (5.5%)	80.2% (24.3%)	60.4% (4.5%)
	Accuracy	Overall	76.2% (12.1%)	83.7% (1.3%)	89.2% (7.0%)	83.7% (0.6%)
		Filtered	15.1% (14.8%)	46.1% (15.9%)	10.6% (13.7%)	52.7% (8.5%)
